# Practical Observations on Hæmorrhage from the Extraction of Teeth, and Dental Fistulæ

**Published:** 1849-07

**Authors:** 


### PRACTICAL OBSERVATIONS ON HÆMORRHAGE FROM THE EXTRACTION OF TEETH, AND DENTAL FISTULÆ.

M. Felix le Gros communicated the following case at a recent session of the "Societé Medicale du Temple." A dentist, M. Bianchi, extracted the first molar tooth in the lower jaw on the right side, for a journeyman butcher, 23 years of age, of a strong constitution. During fourteen hours alarming hæmorrhage continued, against which several general practitioners contended in vain. The patient being conveyed to La Pitié, had the blood staunched for a time, but scarcely had he left the hospital when it broke out afresh, more profuse than before. Dr. Berthier, aided by his nephew, Dr. La Grange, applied the actual cautery seven times in one day. Lastly, all the known means having failed, he was conveyed, in an exhausted state, to the Hotel Dieu, and placed under the care of M. Roux, who, after having heard the preceding details, decided on tying the carotid artery. While preparations were making for the operation, some indiscreet persons having intimated to the patient what was going to be done, he fairly made his escape. It was subsequently ascertained that after a long fainting fit, the hæmorrhage had stopped, but that the young man was subject to profuse bleeding, even from the slightest cut.

M. Perrot, the dentist, it was mentioned, had recently lost one of his patients similarly affected.

M. Segalas also knew a woman who died in this manner.

M. Toirac observed that hæmorrhage, properly so called, supervening on the extraction of a tooth, was generally of short duration, and commonly yielded to the application of acidulated or alcoholic preparations, or to the simple pressure made for a few moments on the gums with the thumb and index finger, to favor the formation of a coagulum, which, like a cork, should mechanically close the alveolus. But it was not always the case, particularly when hæmorrhage, which, instead of showing itself immediately, broke out ten, twenty, or thirty hours after the operation, and that without premonitory symptoms, fracture of the bone, or laceration of the gums. The most energetic astringents, cauterisation, compression with lint, &c., did not always suffice. The means which had succeeded in numerous cases which came under his care, was the use of wax, mentioned by most authors, but it required a certain method of application. That which might be best depended on was purified white wax, which required a high temperature to soften it. It was to be heated until it kneaded easily between the fingers; a piece of it was then placed on the space left vacant by the tooth, and pressed in so as to make it penetrate into all the irregularities of the alveolus. A small roll of linen was next placed over it, thick enough, when the dental arches are in apposition, to compress the wax, and maintain it *in situ.* A chin bandage completed the dressing. This should be kept up for at least two or three hours, and the patient prevented from opening his mouth. Unless compression was made in the manner above mentioned, the piece of wax would be driven out, and valuable time lost. M. Toirac added a few reflections on dental fistulæ. He remarked that they frequently form of themselves an opening in points at some distance from the diseased teeth, which have produced and entertain them. More than twenty years ago he had occasion to open an abscess which developed itself between the lower eye-lid and the globe of the eye. The purulent matter continued to flow until a long neglected stump of the cuspidatus tooth was removed. He had recently attended a foreign prince, who had, when he came to consult him, a tumor in the palate, occupying a considerable space, not very prominent, indolent to the touch, and not affecting the color of the palatine membrane. A few months previously, a tumor had opened in the left nasal fossa. The teeth, examined with care, seemed to be soundly set in a firm rosy gum, with the exception of the left small incisor, on which a slight motion might be impressed by seizing it between the fingers, a symptom sufficient to indicate that the seat of the disease was to be found at the extremity of its root.

Notwithstanding his repugnance to sacrifice this tooth, so beautiful and white, he resolved to extract it. When the operation was finished, a pressure made by the patient on the tumor with the tip of his tongue, caused to flow from the alveolus an almost limpid and slightly colored fluid, such as is frequently met with in cysts, common to the maxillæ. Some warm water, injected through the alveolus by means of a narrow canula, easily found its way into the tumor, and he had every reason to believe that a radical cure would shortly take place. During the following days, however, the tumor continued to fill as fast as it was emptied, and he then decided on making a counter-opening on the side of the palate, by means of a small cautery applied to the part which appeared most prominent. An injection forced through the opening, passed rapidly by the alveolar cavity. The next day the tumor had collapsed, and a few days after entirely disappeared.

As a practical fact, said M. Toirac, I would observe, that in most cases it is proper to open tumors which develope themselves in the palate, with the red hot iron, in order to avoid the hæmorrhage which frequently follows their incision with a cutting instrument, and which, let me add, is very difficult to arrest.

